# Critical Dynamics in Spontaneous Resting-State Oscillations Are Associated With the Attention-Related P300 ERP in a Go/Nogo Task

**DOI:** 10.3389/fnins.2021.632922

**Published:** 2021-03-22

**Authors:** Nadine D. Herzog, Tim P. Steinfath, Ricardo Tarrasch

**Affiliations:** ^1^Department of Neurology, Max Planck Institute for Human Cognitive and Brain Sciences, Leipzig, Germany; ^2^School of Education and Sagol School of Neuroscience, Tel Aviv University, Tel Aviv, Israel

**Keywords:** attention, detrended fluctuation analysis, P300, resting-state EEG, LRTC

## Abstract

Sustained attention is the ability to continually concentrate on task-relevant information, even in the presence of distraction. Understanding the neural mechanisms underlying this ability is critical for comprehending attentional processes as well as neuropsychiatric disorders characterized by attentional deficits, such as attention deficit hyperactivity disorder (ADHD). In this study, we aimed to investigate how trait-like critical oscillations during rest relate to the P300 evoked potential—a biomarker commonly used to assess attentional deficits. We measured long−range temporal correlations (LRTC) in resting-state EEG oscillations as index for criticality of the signal. In addition, the attentional performance of the subjects was assessed as reaction time variability (RTV) in a continuous performance task following an oddball paradigm. P300 amplitude and latencies were obtained from EEG recordings during this task. We found that, after controlling for individual variability in task performance, LRTC were positively associated with P300 amplitudes but not latencies. In line with previous findings, good performance in the sustained attention task was related to higher P300 amplitudes and earlier peak latencies. Unexpectedly, we observed a positive relationship between LRTC in ongoing oscillations during rest and RTV, indicating that greater criticality in brain oscillations during rest relates to worse task performance. In summary, our results show that resting-state neuronal activity, which operates near a critical state, relates to the generation of higher P300 amplitudes. Brain dynamics close to criticality potentially foster a computationally advantageous state which promotes the ability to generate higher event-related potential (ERP) amplitudes.

## Highlights

- Long−range temporal correlations (LRTC) of resting-state brain activity is related to the amplitude of the P300 event-related potential (ERP).
- Behavioral task performance is negatively related to resting-state LRTC.
- Behavioral task performance is positively related to P300 ERP amplitudes and negatively to P300 ERP latencies.

## Introduction

Sustained attention is a cognitive ability with fundamental importance for general cognitive functioning. It can be described as the ability to allocate attentional resources to focus on a task while preserving a constant performance level (Lukov et al., 2015). Sustained attention hence enables us to concentrate on task-relevant information, even in the presence of distracting stimuli, and thus helps us efficiently carry out tasks that take a long time to complete. Understanding the mechanisms underlying this ability is critical for comprehending neuropsychiatric disorders characterized by attentional deficits. One eligible measure to estimate sustained attention abilities is the intra-individual reaction time variability (RTV). As opposed to mean reaction times, RTV captures fluctuations in attention, as it reflects the fact that in some trials subjects react very fast (when they attend), while in others the reaction times are delayed, probably representing lapses in concentration (Slocomb and Spencer, 2009; Vaurio et al., 2009). Importantly, RTV has specifically been linked to impairments of attention (Sonuga-Barke and Castellanos, 2007). As such, various studies have shown that individuals with attention deficit hyperactivity disorder (ADHD) display significantly larger RTV (Leth-Steensen et al., 2000; Castellanos et al., 2005; Nigg et al., 2005; Klein et al., 2006) emphasizing the reliably of this measure.

### Neural Correlates of Sustained Attention

Long-range temporal correlations (LRTC) are an indicator for temporal autocorrelations in the amplitude fluctuations of ongoing neuronal oscillations and were suggested to indicate when neuronal systems operate at a near-critical point (Linkenkaer-Hansen et al., 2001; Nikulin and Brismar, 2004; Palva et al., 2013; Meisel et al., 2017). Criticality in neuronal systems can be defined as a state where the system is optimally balanced between order and disorder. The stronger the LRTC in activity fluctuations, the closer the neuronal system is to the critical point (Poil et al., 2012). Brain dynamics near a critical state were associated with optimal information processing which, when disrupted, could lead to significant loss of function (Linkenkaer-Hansen et al., 2005; Monto et al., 2007; Montez et al., 2009; Nikulin et al., 2012). Interestingly, LRTC were found to have a highly heritable component (Linkenkaer-Hansen et al., 2007) and high test–retest reliability (Nikulin and Brismar, 2004), which makes them an eligible candidate for a marker of inherent network characteristics. Research suggests that LRTC play a vital role in higher-order brain functions, such as working memory (Mahjoory et al., 2019) and attention (Irrmischer et al., 2018). Notably, Palva et al. (2013) showed that higher LRTC in resting-state brain activity are correlated to higher LRTC in behavioral performance fluctuations in a perceptual threshold task. Furthermore, Irrmischer et al. (2018) found that high sensorimotor alpha LRTC during rest predict good performance on a sustained visual attention task. Taken together, these results indicate a crucial role of LRTC during rest in attentional processes.

Moreover, attentional processes have frequently been linked to the event-related potential (ERP) P300 component (Polich, 2007). This component reflects a positive peak inflection in the brain potential at approximately 250–500 ms after a target stimulus is presented (Polich, 2007). The P300 is traditionally assessed using an “oddball paradigm,” in which subjects are presented with a sequence of stimuli, which are occasionally interrupted by a divergent target stimulus that the subject is expected to detect (Polich and Kok, 1995). The amplitude of the P300 is proportional to the level of attentional resources engaged in processing a given stimulus, and has consistently been reported to be decreased in disorders involving attentional impairments (e.g., ADHD; Johnstone et al., 2013; Tye et al., 2014).

The literature hence suggests that sustained attention abilities can be related to trait measures such as LRTC, as well as evoked measures such as ERP’s. However, whether trait-like oscillatory activity directly influences or even generates (attention-related) P300 is still under debate (e.g., Klimesch et al., 2006, 2007; Sauseng et al., 2007). In the present study, we therefore aim to investigate this relationship. We expect to replicate the positive relationship between P300 and sustained attention, in that higher P300 amplitude, as well as earlier peak latencies, will be related to better sustained attention performance. Furthermore, we predict a positive correlation between resting LRTC and sustained attention performance. Finally, we hypothesize that stronger LRTC during rest will be related to higher P300 amplitude. To test these hypotheses, we recorded 5 min eyes-closed resting-state EEG to calculate LRTC in different frequency ranges, followed by a continuous performance test (CPT, using the oddball paradigm mentioned above) during which EEG was recorded to extract P300 ERPs.

## Materials and Methods

### Participants

The study included data from two participant cohorts. All participants underwent the same task under the same conditions. Dataset 1 consisted of data from 15 healthy participants. Dataset 2 consisted of data from 30 participants that were recruited through the students’ dean’s office (center for students with learning disabilities), or via flyers that were posted in Tel Aviv University, calling for participants that experience attentional problems, to ensure variation in RTV. The whole dataset hence consisted of data from 45 participants. The original study design included a mindfulness intervention, where participants were allocated into intervention vs. waiting-list control groups. In the present work, however, we used the pre-workshop data only. Exclusion criteria were a history of stroke, brain injuries, or neurological problems. All participants were debriefed about the experimental procedure before the experiment, through signed informed consent forms. All participants had a normal or corrected-to-normal vision.

### Experimental Design

At first, 5 min eye-closed rest EEG was recorded. Then, participants completed the CPT. Additional tests, not used in the present study, were also conducted. Each measurement session lasted approximately 2 h. Stimuli were generated on an HP Compaq 8000 Elite computer running OpenSesame version 2.9.2 and presented against a gray background on a Samsung SyncMaster 2233RZ 120 Hz screen. The viewing distance to the screen was 70 cm. Participants performed the tasks while seated in a separate experiment room.

### Attention Assessment

In order to assess sustained attention, we used a CPT, which is a computer-based test. Participants were presented with a 12-min-long sequence of repetitive stimuli that had several different forms (square, star, triangle, and circle) and colors (red, blue, green, and yellow). They were instructed to respond to a single re-occurring pre-specified target (red square) by pressing the spacebar. The target stimulus occurred in 30% of the trials. The inter-trial interval was variable with an average of 2 s. Responses to all other non-target stimuli had to be inhibited. Accuracy and RTV were calculated from the collected data. Accuracy was defined as the proportion of correct trials. Prior to calculating RTV metrics, error trials, defined as very fast or slow (≥3 SDs below or above the individual mean RT) reaction times were removed. RTV was then calculated as the SD of correct target RT (see also: Saville et al., 2015).

### EEG Recordings

EEG data were recorded using a BioSemi Active Two system with 64 Ag/AgCl electrodes. The electrodes were arranged on a nylon cap according to the international standard 10-20 system for electrode placement. Eye movements were monitored using three additional electrooculography (EOG) electrodes, with bipolar horizontal electrodes placed at the outer canthi of each eye, and one EOG electrode placed below the right eye. Two reference electrodes were placed on the mastoids. Data were collected at a sampling rate of 1024 Hz.

### EEG Data Processing

EEG data were processed using Matlab R2015b software in conjunction with the EEGLAB toolbox (Delorme and Makeig, 2004). EEG data were downsampled to 256 Hz and filtered using a Hamming windowed sinc finite impulse response filter with a high-pass frequency of 1 Hz. Line noise at 50 Hz was reduced using the cleanline EEGLAB plugin (Mullen, 2012) and subsequently low-pass filtered at 42 Hz to omit frequencies contaminated with residual line-noise artifacts. The ongoing EEG signal was visually inspected and on average 9.1% of the resting-state data were removed due to transient artifacts. Noisy channels were removed from the data [average channels removed: 1.7 (SD = 1.6) for resting-state and 1.2 (SD = 2.1) for CPT-task data].

The data were re-referenced to the common average and independent component analysis (ICA) was performed using the extended Infomax algorithm (Delorme et al., 2007). The resulting components were visually inspected, and artifactual components related to eye movements, heartbeat, or muscle were removed from the data [average components removed: 6 (SD = 3) for resting-state, and 9 (SD = 6) for CPT-task data]. Removed channels were interpolated using spherical interpolation after the ICA component rejection.

Long-range temporal correlations were calculated using detrended fluctuation analysis (DFA). First, EEG signals were filtered into the classical frequency bands (delta 1–4 Hz, theta 4–8 Hz, alpha 8–13 Hz, beta 13–30 Hz, and gamma 30–45 Hz). The amplitude envelope was extracted for each frequency band using the Hilbert transform. Then, the cumulative sum of the signal was calculated and subsequently integrated and linearly detrended. Finally, the root-mean-square fluctuation was calculated as a function of window size and plotted on double logarithmic axes. The following time window ranges were used for the different frequency bands: 5–30 s for delta and theta, 2–30 s for alpha, and 1–30 s for beta and gamma. Different lengths of the shortest windows were used to avoid autocorrelations introduced by filtering the data. Fitting a least-squares line eventually gives the DFA exponent, as reflected by the slope of the line (see Hardstone et al., 2012 for further details).

The P300 ERP was calculated using event-related data from the CPT recordings. First, the data were segmented into epochs, starting 200 ms before the stimulus presentation and ending 800 ms after it. The epochs were visually inspected, and noisy epochs were rejected. Subsequently, the data were sorted into bins corresponding to target and non-target stimuli. On average, 54 (*SD* = 17) epochs corresponding to the target stimulus and 122 (*SD* = 43) epochs for non-target stimuli were retained for each subject. The epochs were baseline corrected using 100 ms pre-stimulus data. ERPs for each subject were then computed by averaging the trials in the target and non-target bin. ERP latencies were computed as the duration from stimulus onset to the peak amplitude in the window of 250–600 ms after stimulus onset. ERP amplitudes were calculated as the mean amplitude in this window.

**FIGURE 1:**
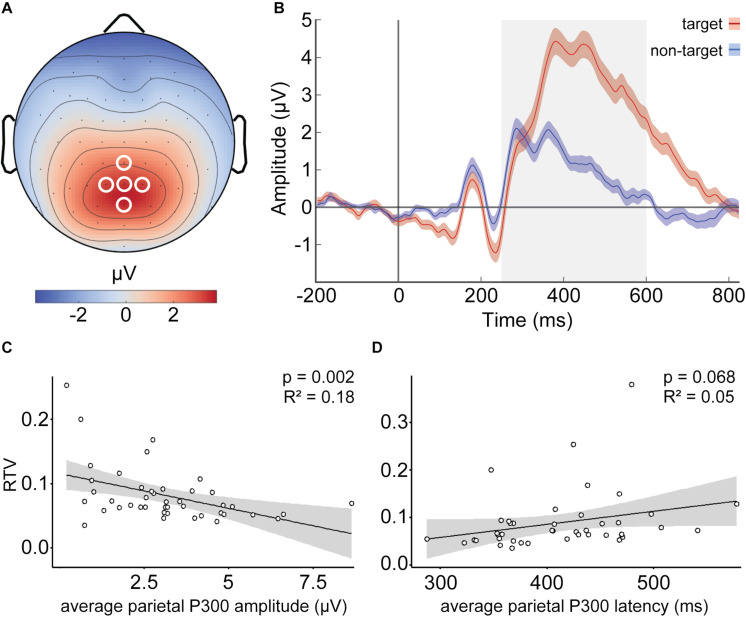
Parietal target P300 amplitude predicts reaction time variability (RTV). **(A)** Group average scalp topography of event-related potentials (ERPs) in response to target stimuli; white circles indicate parietal electrodes (Pz, CPz, POz, P1, and P2) used for average ERP calculation. **(B)** Group-level average ERPs related to target (red) and non-target (blue) stimuli with standard error of the mean as shaded area. Gray area indicates the time window (250–600 ms) used for mean P300 amplitude and peak latency calculation. **(C)** Lower target P300 amplitudes are associated with higher RTV. **(D)** A trend toward significant association between target P300 latencies and RTV was observed. Each dot in **(C,D)** represents each participant’s average.

Since P300 activity—especially in the context of target detection—is expected to be mainly elicited in parietal electrodes (Polich, 1986), we chose a parietal cluster including Pz and its adjacent electrodes CPz, P1, P2, and POz for our analyses (Figure 1A). Target ERPs in these electrodes were averaged, producing a parietal average ERP. All further analyses were based on this parietal average target ERP. For control analyses, we also calculated non-target P300 ERPs, which were calculated as described above, except that the data were segmented into bins corresponding to onsets of non-target stimuli. Target and non-target ERP waves are depicted in Figure 1B.

## Results

The CPT average accuracy was 97.1% (*SD* = 0.057, *min* = 69.79, *max* = 100), while all subjects performed above chance (>50% of possible hits). The mean reaction time was 445 ms (*SD* = 0.094 ms, min = 335 ms, max = 855 ms) and the average RTV was 0.088 s (SD = 0.062 s, min = 0.035 s, max = 0.378 s). The P300 ERP group average amplitude was 3.191 μV (*SD* = 1.753, *min* = 0.215, *max* = 8.639) and group average peak latency was 411.689 ms (*SD* = 70.175, *min* = 251.302, *max* = 601.562). Overall, grand average DFA (average across all participants and all electrodes) was in accordance with previously reported values (Table 1).

**TABLE 1:** Group average DFA exponents.

Frequency	Mean (SD)	Min	Max
Delta	0.597 (0.027)	0.543	0.673
Theta	0.665 (0.094)	0.527	0.877
Alpha	0.750 (0.079)	0.629	0.948
Beta	0.725 (0.074)	0.614	0.939
Gamma	0.677 (0.067)	0.565	0.839

### ERP Predicts RTV

To investigate whether the P300 amplitude predicts RTV, we built a linear regression model using the lm() function in R Studio (Version 1.2.5042), with average parietal P300 amplitude as the predictor. Assessment of regression assumptions revealed an influential case with a Cooks distance greater than 3, which was also defined as an outlier in the Q–Q plots, that led to non-linearity of the standardized residuals. After the removal of this case, assumptions for regression were met. RTV showed a significant negative association with P300 amplitude (β = −0.451 μV/RTV, *SE* = 0.003, *p* = 0.002, *adjusted R*^2^ = 0.18), indicating that higher parietal P300 amplitude was related to lower RTV (Figure 1C). To test whether P300 latency predicts RTV, we built a second linear model, this time with RTV as the outcome and average parietal P300 latencies as the predictor. Inspection of Q–Q plots and standardized residuals for each model indicated that the assumptions for regression were met. RTV showed only trend-significant association with P300 latency (β = 277, SE = 149.14, *p* = 0.068, *adjusted R*^2^ = 0.05) (Figure 1D).

### DFA Predicts RTV

To test the relationship between DFA and RTV, we performed separate regression analyses for each channel. Statistical significance of the results was assessed using a cluster-based permutation approach controlling for multiple comparisons (Maris and Oostenveld, 2007). Clusters were defined as two or more neighboring electrodes which showed a significant (*p* < 0.05) association between DFA and RTV. We generated a permutation distribution by randomly shuffling the RTV data across subjects and repeating the regression analysis 1000 times. For each permutation, new significant clusters were identified and the summed *t*-value of all electrodes included in these clusters was saved. Results were considered significant when the summed *t*-value of real clusters was greater than 95% of the summed *t*-values of randomly formed clusters based on the permuted data.

**FIGURE 2:**
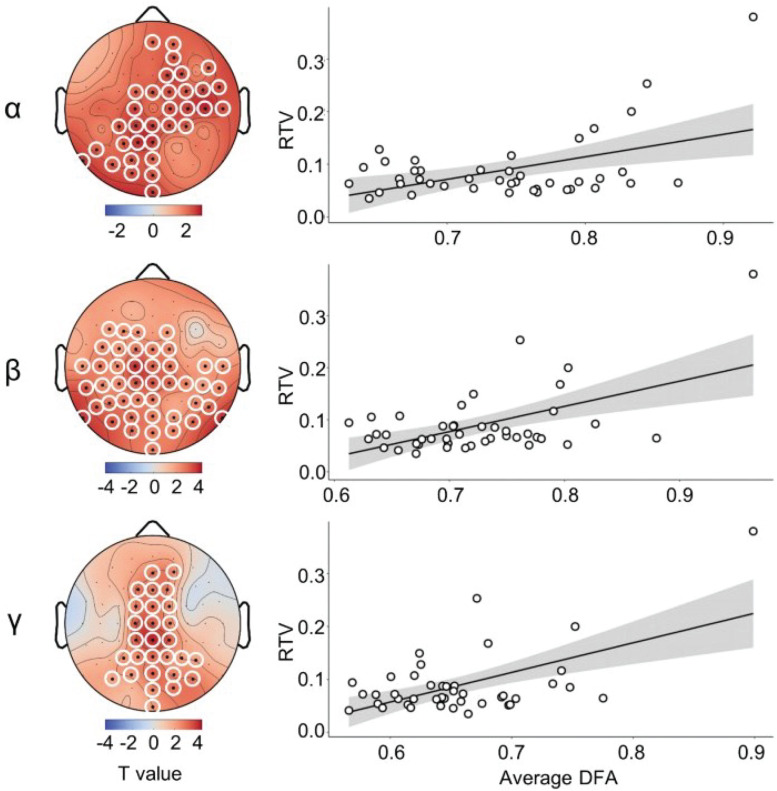
DFA predicts RTV. **Left panel** shows topographies of significant clusters in the alpha, beta, and gamma frequency range. **Right panel** shows the respective scatterplots, illustrating the relationship between RTV and the average DFA across electrodes in the cluster.

Results showed positive relationships between RTV and DFA in alpha, beta, and gamma frequency range, thus indicating that higher scaling exponents during rest were related to higher RTV. Scalp topographies of the regression coefficients are depicted on the left panel of Figure 2. The right panel of Figure 2 shows scatterplots for RTV against the cluster wise average scaling exponent across all participants.

In the *delta* frequency, 10 electrodes showed a significant association with RTV. Due to spatial adjacency, two clusters were built from eight out of these electrodes. These clusters did not reach significance after permutation testing (*p* > 0.05, data not shown). As for LRTC in the *theta* frequency band, we found three electrodes that significantly predicted RTV, two of which were included in the to be tested cluster. This cluster, however, did not reach significance after permutation testing (*p* > 0.05, data not shown). LRTC in the *alpha* frequency resulted in one cluster of 34 channels distributed above right fronto-temporal and left parieto-occipital areas (Figure 2, left), which was significant after permutation testing (*p* < 0.01). In the *beta* frequency range, the spatial distribution of the significant regression coefficients was rather broad, resulting in one cluster including 44 electrodes. This cluster was significant at *p* < 0.01. As for LRTC in the *gamma* frequency range, we found one cluster of 28 electrodes that significantly predicted RTV. This cluster was mainly above the central line and parietal cortex and remained significant at *p* < 0.001 after permutation testing.

### DFA Correlates Positively With ERP Amplitude

**FIGURE 3:**
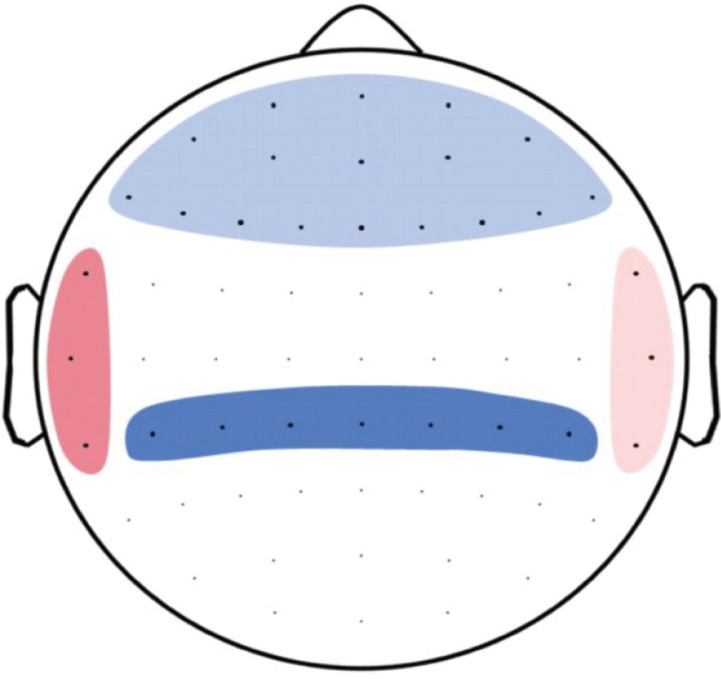
Topoplot of DFA clusters used to predict P300. Frontal cluster included the channels Fp1, Fpz, Fp2, AFz, AF3, AF7, AF4, AF8, Fz, F1-8, FCz, and FC1-6 (light blue). Temporal right cluster included channels TP8, T8, and FT8 (light red). Temporal left cluster included channels TP7, T7, and FT7 (dark red), and the inferior parietal cluster included channel CP1-5 and CPz (dark blue).

To test the relationship between LRTC and the P300 potential, we build hypothesis-driven clusters of electrodes to reduce multiple testing. Because the P300 potential is thought to have its generators in inferior parietal, temporal, and prefrontal regions (Polich, 2007), DFA clusters were built according to electrodes that most likely capture activity in these regions using findings by Koessler et al. (2009) as a reference. Clusters were as follows: inferior parietal cluster—channels CP1-5 and CPz; temporal left—channels TP7, T7, FT7; temporal right—channels TP8, T8, FT8; and frontal cluster—channels Fp1, Fpz, Fp2, AFz, AF3, AF7, AF4, AF8, Fz, F1-8, FCz, FC1-6 (see Figure 3). We then calculated separate linear regression models, including average parietal P300 as the outcome and average DFA of each cluster as the predictor. We corrected for multiple comparisons using the binomial multiple-comparison method (Montez et al., 2009; Poil et al., 2011; Irrmischer et al., 2018). This method tests whether a significant number of clusters reach the significance level of *p* < 0.05. According to the binomial distribution, the likelihood of having five out of 20 significant results (see below) by chance is <5% (Montez et al., 2009; Nikulin et al., 2012; Schiavone et al., 2014). Analysis of standardized residuals was carried out for each model to identify outliers. None of the cases had an undue influence [all cases showed a Cook’s distance (CD) < 1]. Inspection of Q–Q plots and standardized residuals for each model indicated that the assumptions for regression were met.

None of the clusters showed a significant association with P300 amplitude (Supplementary Table S1).

**FIGURE 4:**
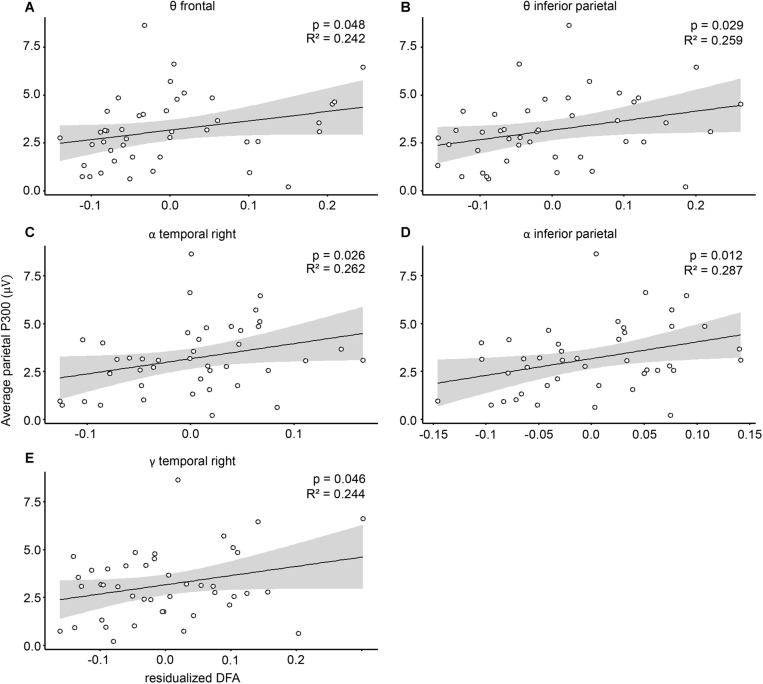
Cluster DFA predict P300 amplitude. Associations between P300 mean amplitude and residualized DFA in the **(A)** theta frequency in frontal, **(B)** theta frequency in inferior parietal, **(C)** alpha frequency in temporal right, **(D)** alpha frequency in interior parietal, and **(E)** gamma frequency in temporal right cluster. P300 amplitudes are plotted against the residualized DFA values, controlling for the covariation with reaction time variability.

Because LRTC, as well as P300, are both linked to RTV (as shown above), we suspected that individual sustained attention abilities may mask a potential relationship between P300 and LRTC. We therefore repeated the analysis, this time including RTV as a covariate. Inspection of CD indicated that one of the cases had a high influence (CD > 3 in all 20 models). This case was also an outlier in the Q–Q plots and led to non-linearity of the standardized residuals. After the removal of that case, regression assumptions regarding linearity and homoscedasticity were met for all the models. Next, because we previously observed a relationship between DFA and RTV, we tested whether the predictors RTV and cluster DFA violated the assumption of multicolinearity, using the variance inflation factor (VIF; Akinwande et al., 2015). VIF was around 1 for all of the models, indicating low correlations among predictors. The final results showed that five out of the 20 DFA clusters significantly predicted average parietal ERP P300 amplitude (*binom. p* = 0.002). Among these were the DFA in the theta band in frontal and inferior parietal clusters (Figures 4A,B and Supplementary Table S2), as well as in the alpha band in inferior parietal and temporal right clusters (Figures 4C,D and Supplementary Table S2), and the gamma band in a temporal right cluster (Figure 4E and Supplementary Table S2).

All associations were positive, indicating that higher LRTC are related to higher P300 amplitudes. The DFA from all other clusters and frequency bands did not show significant associations with P300 amplitude (Supplementary Table S2). In terms of P300 latency, our results showed that none of the clusters significantly predicted the P300 peak latency (*p* > 0.05 for all electrodes). When controlling for RTV, two clusters in the alpha range (frontal and temporal right) were significant. However, this significance does not survive multiple comparison correction (*binom. p* = 0.189; Supplementary Table S3). To investigate whether this effect is specific, we also tested whether non-target standard stimuli P300 could likewise be predicted by DFA. Results show that none of the DFA clusters was significantly associated with non-target ERP (Supplementary Table S4).

## Discussion

In this study, we aimed to establish how trait-like critical oscillations during rest can predict the attention-related P300 evoked potential. We found that, after controlling for individual sustained attention abilities, LRTC were positively associated with P300 amplitudes but not latencies. This effect was specific to target P300. Furthermore, in line with previous findings, we found that good performance in a sustained attention task was related to higher P300. Unexpectedly, we did not observe a negative, but a positive relationship between RTV and LRTC in ongoing oscillations during rest.

### P300 Predicts RTV

The P300 amplitude is believed to reflect the amount of attentional capacity, while the P300 latency seems to index the speed of attentional processing (Polich and Criado, 2006). Our results hence suggest that the more attentional capacity is allocated to a stimulus (indicated by greater P300 amplitude) the better the sustained attention (as shown by lower RTV). Our results replicate previous findings indicating the validity of our measures. However, while peak latency is the most widely used measure for ERP component latency, it is also easily influenced by noise which is superimposed on the ERP (Kiesel et al., 2008; Liesefeld, 2018). Our results regarding the latency should hence be interpreted with caution and need to be replicated with more robust latency estimation approaches.

### LRTC During Rest Predict RTV

Our results furthermore demonstrate that resting-state LRTC in the alpha, beta, and gamma frequency range were positively associated with sustained attention indexed as RTV. It has previously been suggested that a reduction in DFA exponents indicates reduced autocorrelation within the signal and therefore, less influence of preceding neuronal events on neuronal activation (Gilden, 2001; Irrmischer et al., 2018). In line with this, Irrmischer et al. (2018), for example, argue that lower LRTC may hence be associated with fewer distractions from the focused task. In our data, strong LRTC may reflect more influence on future dynamics of the signal and therefore more potential for the processing of task-irrelevant distracting information, resulting in higher RTV.

Nevertheless, our results seem to be in contrast with previously published results. As such, Irrmischer et al. (2018) and Mahjoory et al. (2019) found higher LRTC during rest to be associated with better sustained and phasic attention abilities, respectively. One possible reason for this contradiction could be that, in both studies, data were collected from a healthy sample, while in our study the sample contained participants with self-reported attention problems. It could be that, in healthy subjects, high resting-state LRTC are advantageous for task performances, while this relationship changes in subjects with attentional problems, where too high LRTC can foster impairments. Indeed, high LRTC, especially in the beta band, have been shown to be associated with pathologies such as epilepsy (Monto et al., 2007). Furthermore, while a DFA of approximately 1 is thought to relate to an optimal ratio of inhibitory and excitatory connections, the exact ratio at this point is not clear (Poil et al., 2012). This could implicate, that in certain cases, a high exponent may reflect a disadvantageous configuration of the network. If we consider criticality in neural systems as a measure for the efficacy of cortical signal propagation (see section “Introduction”), this may not be restricted to the efficacy of task-relevant signal transmission. It could indeed be that the brain of subjects with attentional deficits operates near criticality, but that the information that is processed optimally is task-irrelevant. In line with this, Wilting and Priesemann (2019) argued that criticality may also foster disadvantages and maximize properties that can be adversarial to cortical function. Unfortunately, our data do not allow to test whether our suggested relationship is true. Future studies may account for this question by ensuring a wide range of RTV, possibly via the inclusion of, for example, patients diagnosed with attention disorders (extreme bad end of sustained attention) to trained meditators (extreme good end of sustained attention).

Another explanation might be deduced from studies which indicate that sustained attention performance correlates with the participants’ ability to suppress LRTC from rest to task (Irrmischer et al., 2018). Consequently, in our sample, due to overly high resting LRTC, the observed attentional impairments might be a consequence of the inability to suppress LRTC when changing the demands from rest to task.

Notably, we also observed a positive relationship between beta and gamma resting-state LRTC and RTV. Both frequencies are implicated in attentional processes and interact in complex ways (e.g., Bauer and Pllana, 2014; Richter et al., 2017). As such, a study by Bastos et al. (2018) showed that beta oscillations originating in deep cortical layers modulate gamma activity in superficial layers. This would also be in line with the topographies observed in our data, where beta had a wide-spread distribution, while gamma effects were specifically restricted to midline electrodes. However, adequate source-modeling would be necessary to validate this assumption.

### LRTC During Rest Predict P300 Amplitude

Lastly, we observed a positive association between target P300 amplitude and theta LRTC in frontal and parietal, alpha LRTC in parietal and temporal right areas, and gamma LRTC in temporal right areas. These effects are specific to the P300 potential elicited upon target stimulus presentation as no such associations were observed for non-target stimuli. These findings add to previous research as they show that not only spatially distributed resting-state networks exert an influence on P300 amplitudes (Li et al., 2015, 2020), but also that the temporal structure of the oscillations is important.

Frontal theta, as well as parietal alpha power, has frequently been associated with cognitive control processes (van Driel et al., 2012; Popov et al., 2018) and with the fronto-parietal attention network (Ptak, 2012). Moreover, these areas have previously been associated with P300 amplitude in the processes of attention allocation (Daffner et al., 2003; Li et al., 2015). Our data demonstrate that the brain areas involved not only need to work together and in synchrony (Zhang et al., 2014), but also close to a critical state, in order to modulate the generation of the P300 ERP in a beneficial way. It could be that these critical dynamics foster a computationally advantageous state, which promotes the ability to generate higher ERP amplitudes. LRTC are usually found to be reduced in clinical samples [e.g., in depression (Linkenkaer-Hansen et al., 2005) or schizophrenia (Nikulin et al., 2012)]. These observed attenuations of LRTC are assumed to be due to increased variability in neuronal activity, indicating less efficient information processing. This could explain why the amplitudes of the P300 are lower with lower LRTC: The network does not process information efficiently enough, thus leading to less additive and/or phase-resetting effects that are necessary to generate P300 amplitudes.

Against our expectations, however, this proposed mechanism does not seem to relate directly to sustained attention, as the relationship between resting LRTC and ERP only holds when controlling for sustained attention abilities. One explanation could be that lower frequency oscillations, such as theta, rather prepare neural systems for the processing of information (Klimesch et al., 2007; Jensen et al., 2010; Mathewson et al., 2011; Spitzer and Haegens, 2017), while other down-stream processes affect the final behavioral output (Womelsdorf et al., 2010; Cavanagh and Frank, 2014). In line with this, specifically frontal theta was shown to be associated with the initial organization of relevant information rather than direct behavioral output (Womelsdorf et al., 2010; Cavanagh and Frank, 2014).

The fact that we did not observe an association between LRTC and P300 latency is in line with a study reporting connectivity between frontal and parietal areas to be predictive for ERP amplitude but not latency (Li et al., 2015). Since the P300 peak latency is prone to be influenced by noise (Li et al., 2015), our estimations should therefore be interpreted with caution.

Overall, our findings support the notion that trait-like critical resting-state dynamics foster the generation of event-related P300 amplitudes.

### Limitations and Drawbacks

One limitation of the current study is the mixed nature of our sample containing subjects with various degrees of self-reported attentional problems as well as healthy subjects. However, our results indicate that it is valuable to further investigate the relationship of resting-state LRTC, P300, and RTV in a better classified sample, for example, including subjects diagnosed with ADHD.

Furthermore, while LRTC in the alpha and beta frequency range are generally a stable measure as indicated by high test–retest reliabilities across recording sessions (Nikulin and Brismar, 2004), the correlations with the gamma frequency DFA should be treated with caution, since the stability in this frequency range has not been established yet.

Lastly, we would like to note that one should be cautious to make strong inferences regarding the relationship we observe between LRTC and RTV. Although we present possible explanations for the observed effects, our findings are in contrast to previous studies (Simola et al., 2017; Irrmischer et al., 2018; Mahjoory et al., 2019) and seem suspicious in the context of the other results we observe. We found that higher LRTC predicted higher P300 amplitudes, and higher P300 amplitudes predicted better sustained attention. Consequently, one would expect to find that higher LRTC would likewise predict better sustained attention abilities, thus closing the loop. This is not the case in our data.

### Overall Conclusion

Our results indicate that network dynamics operating close to critical state foster the generation of task-relevant activity as is reflected in the generation of larger task evoked P300 amplitudes. However, in our sample, this relationship can only be observed for “normalized” networks, where the inter-individual variability related to differences in attentional abilities is accounted for. It is hence conceivable that by adding RTV as a covariate to the model, we account for variance that explains attention ability in the P300 that is unrelated to LRTC. This would also be in line with studies showing that LRTC in response time fluctuations do not relate to RTV, but to commission errors only (Simola et al., 2017). Variations in sustained attention abilities may thus mask the relationship between LRTC and the P300 amplitude, implicating that LRTC rather modulate other factors related to P300 generation, such as response selection or executive processes. Our data, unfortunately, do not allow to test this hypotheses, and future studies may account for this.

### Importance of Results

P300 is a widely used biomarker for evaluating potential cognitive impairments and has substantial application in clinical diagnosis (Polich, 2007; Howe et al., 2014; Cecchi et al., 2015; Turetsky et al., 2015) and cognitive neuroscience (Sellers and Donchin, 2006; Nijboer et al., 2008). However, there is a large variability in P300 across and within subjects, emphasizing the importance to identify the underlying neural mechanisms that foster this variation. The data of the present study contribute to the existing literature by showing that the complex network interactions from which P300 emerges, require to operate near a critical state in order to generate higher P300 amplitudes.

## Data Availability Statement

The data that supports the findings of this study are available upon request from the corresponding author NH (naherzog@cbs.mpg.de). The Data cannot be made publicly available due to the privacy policies for human biometric data according to the European General Data Protection Regulation (GDPR).

## Ethics Statement

The studies involving human participants were reviewed and approved by the Tel Aviv University Ethics Committee. The patients/participants provided their written informed consent to participate in this study.

## Author Contributions

NH: data acquisition/investigation, conceptualization, formal analysis, visualization, writing—original draft, and writing—review and editing. TS: conceptualization, formal analysis, visualization, writing—original draft, and writing—review and editing. RT: conceptualization, resources, supervision, funding acquisition, and writing—review and editing. All authors contributed to the article and approved the submitted version.

## Conflict of Interest

The authors declare that the research was conducted in the absence of any commercial or financial relationships that could be construed as a potential conflict of interest.

## Supplementary Material

Supplementary Table 1 P300 amplitude predicted by DFA clusters.

Supplementary Table 2 P300 amplitude predicted by DFA clusters and reaction time variability.

Supplementary Table 3 P300 latency predicted by DFA clusters and reaction time variability.

Supplementary Table 4 Non-target P300 amplitude predicted by DFA clusters and reaction time variability.
