# Supplementary material for: Critical Dynamics in Spontaneous Resting-State Oscillations Are Associated With the Attention-Related P300 ERP in a Go/Nogo Task
Source: Front Neurosci. 2021 Mar 22;15:632922. doi: 10.3389/fnins.2021.632922 (PMC8019703; doi:10.3389/fnins.2021.632922)
Supplement: Supplementary Table 1 — P300 amplitude predicted by DFA clusters. [file Data_Sheet_1.docx]

**SUPPLEMENTARY** **MATERIAL**

| Table S1. P300 amplitude predicted by DFA clusters | | | | |  | |  | |
| --- | --- | --- | --- | --- | --- | --- | --- | --- |
| **cluster** | ***p*** | | **adj. R²** | **stand. beta** | | **SE** | |  |
| delta |  |  |  |  | |  | |  |
| inferior parietal |  | 0.266 | 0.006 | 0.169 | | 7.939 | |  |
| frontal |  | 0.567 | -0.015 | -0.088 | | 9.621 | |  |
| temporal right |  | 0.769 | -0.021 | -0.045 | | 5.598 | |  |
| temporal left |  | 0.276 | 0.005 | 0.166 | | 4.689 | |  |
| theta |  |  |  |  | |  | |  |
| inferior parietal |  | 0.101 | 0.040 | 0.248 | | 2.318 | |  |
| frontal |  | 0.117 | 0.034 | 0.237 | | 2.548 | |  |
| temporal right |  | 0.337 | -0.001 | 0.147 | | 2.589 | |  |
| temporal left |  | 0.254 | 0.008 | 0.174 | | 2.964 | |  |
| alpha |  |  |  |  | |  | |  |
| inferior parietal |  | 0.064 | 0.056 | 0.279 | | 3.457 | |  |
| frontal |  | 0.545 | -0.014 | 0.093 | | 2.929 | |  |
| temporal right |  | 0.155 | 0.024 | 0.216 | | 3.542 | |  |
| temporal left |  | 0.253 | 0.008 | 0.174 | | 3.455 | |  |
| beta |  |  |  |  | |  | |  |
| inferior parietal |  | 0.184 | 0.018 | 0.202 | | 3.510 | |  |
| frontal |  | 0.265 | 0.006 | 0.170 | | 3.272 | |  |
| temporal right |  | 0.171 | 0.021 | 0.208 | | 3.780 | |  |
| temporal left |  | 0.159 | 0.023 | 0.214 | | 3.406 | |  |
| gamma |  |  |  |  | |  | |  |
| inferior parietal |  | 0.298 | 0.003 | 0.159 | | 3.879 | |  |
| frontal |  | 0.110 | 0.036 | 0.242 | | 3.648 | |  |
| temporal right |  | 0.088 | 0.044 | 0.257 | | 2.556 | |  |
| temporal left |  | 0.330 | -0.001 | 0.149 | | 2.666 | |  |

Table S2. P300 amplitude predicted by DFA clusters and reaction time variability

| **cluster** |  | ***p* cluster** | **adj. R²** | **stand. beta cluster** | **SE** | **stand. beta RTV** | **SE** |
| --- | --- | --- | --- | --- | --- | --- | --- |
| delta |  |  |  |  |  |  |  |
| inferior parietal |  | 0.199 | 0.198 | 0.181 | 7.396 | -0.460 | 5.748 |
| frontal |  | 0.629 | 0.169 | -0.069 | 9.221 | -0.444 | 5.877 |
| temporal right |  | 0.739 | 0.166 | -0.047 | 5.566 | -0.449 | 5.860 |
| temporal left |  | 0.186 | 0.200 | 0.186 | 4.415 | -0.464 | 5.748 |
| theta |  |  |  |  |  |  |  |
| **inferior parietal** |  | **0.029*** | **0.259** | **0.304** | **2.185** | **-0.494** | **5.574** |
| **frontal** |  | **0.048*** | **0.242** | **0.275** | **2.392** | **-0.480** | **5.610** |
| temporal right |  | 0.261 | 0.190 | 0.159 | 2.540 | -0.462 | 5.783 |
| temporal left |  | 0.171 | 0.202 | 0.193 | 2.810 | -0.464 | 5.738 |
| alpha |  |  |  |  |  |  |  |
| **inferior parietal** |  | **0.012*** | **0.287** | **0.348** | **3.321** | **-0.508** | **5.484** |
| frontal |  | 0.233 | 0.193 | 0.171 | 2.793 | -0.485 | 5.877 |
| **temporal right** |  | **0.026*** | **0.262** | **0.314** | **3.413** | **-0.519** | **5.637** |
| temporal left |  | 0.168 | 0.203 | 0.194 | 3.326 | -0.467 | 5.743 |
| beta |  |  |  |  |  |  |  |
| inferior parietal |  | 0.087 | 0.223 | 0.240 | 3.705 | -0.481 | 5.693 |
| frontal |  | 0.225 | 0.194 | 0.171 | 3.238 | -0.460 | 5.762 |
| temporal right |  | 0.114 | 0.215 | 0.222 | 3.706 | -0.467 | 5.694 |
| temporal left |  | 0.104 | 0.218 | 0.228 | 3.457 | -0.468 | 5.684 |
| gamma |  |  |  |  |  |  |  |
| inferior parietal |  | 0.214 | 0.196 | 0.176 | 4.003 | -0.468 | 5.774 |
| frontal |  | 0.071 | 0.230 | 0.251 | 3.576 | -0.462 | 5.631 |
| **temporal right** |  | **0.046*** | **0.244** | **0.277** | **2.335** | **-0.465** | **5.580** |
| temporal left |  | 0.286 | 0.187 | 0.151 | 2.465 | -0.452 | 5.778 |

Table S3. P300 latency predicted by DFA clusters and reaction time variability

| **cluster** |  | ***p* cluster** | **adj. R²** | **stand. beta cluster** | **SE** | **stand. beta RTV** | **SE** |
| --- | --- | --- | --- | --- | --- | --- | --- |
| delta |  |  |  |  |  |  |  |
| inferior parietal |  | 0.893 | 0.032 | -0.021 | 282.261 | 0.281 | 152.640 |
| frontal |  | 0.549 | 0.041 | -0.093 | 343.281 | 0.301 | 155.293 |
| temporal right |  | 0.611 | 0.038 | -0.078 | 207.240 | 0.291 | 152.675 |
| temporal left |  | 0.283 | 0.059 | -0.164 | 166.308 | 0.307 | 151.354 |
| theta |  |  |  |  |  |  |  |
| inferior parietal |  | 0.410 | 0.048 | -0.129 | 86.022 | 0.313 | 155.782 |
| frontal |  | 0.456 | 0.045 | -0.115 | 93.271 | 0.304 | 153.985 |
| temporal right |  | 0.485 | 0.044 | -0.109 | 94.850 | 0.306 | 155.550 |
| temporal left |  | 0.645 | 0.037 | -0.071 | 107.097 | 0.291 | 153.485 |
| alpha |  |  |  |  |  |  |  |
| inferior parietal |  | 0.114 | 0.090 | -0.251 | 129.496 | 0.368 | 156.812 |
| **frontal** |  | **0.046*** | **0.123** | **-0.309** | **101.436** | **0.370** | **150.647** |
| **temporal right** |  | **0.007***** | **0.194** | **-0.420** | **124.160** | **0.426** | **147.320** |
| temporal left |  | 0.124 | 0.087 | -0.238 | 122.651 | 0.341 | 152.206 |
| beta |  |  |  |  |  |  |  |
| inferior parietal |  | 0.888 | 0.033 | -0.024 | 137.648 | 0.288 | 167.808 |
| frontal |  | 0.236 | 0.065 | -0.186 | 118.282 | 0.334 | 155.685 |
| temporal right |  | 0.490 | 0.043 | -0.109 | 139.440 | 0.311 | 157.702 |
| temporal left |  | 0.292 | 0.058 | -0.169 | 127.343 | 0.339 | 159.590 |
| gamma |  |  |  |  |  |  |  |
| inferior parietal |  | 0.675 | 0.036 | -0.069 | 146.954 | 0.304 | 163.351 |
| frontal |  | 0.184 | 0.073 | -0.205 | 134.058 | 0.331 | 152.978 |
| temporal right |  | 0.208 | 0.069 | -0.189 | 90.249 | 0.293 | 148.528 |
| temporal left |  | 0.537 | 0.041 | -0.093 | 93.271 | 0.280 | 150.288 |

Table S4. Non-target P300 amplitude predicted by DFA clusters and reaction time variability

| **cluster** |  | ***p***  **cluster** | **adj. R²** | **stand. beta cluster** | **SE** | **stand. beta RTV** | **SE** |
| --- | --- | --- | --- | --- | --- | --- | --- |
| delta |  |  |  |  |  |  |  |
| inferior parietal |  | 0.679 | 0.044 | -0.063 | 4.390 | -0.281 | 2.374 |
| frontal |  | 0.055 | 0.123 | -0.291 | 5.134 | -0.217 | 2.323 |
| temporal right |  | 0.438 | 0.054 | -0.118 | 3.215 | -0.271 | 2.369 |
| temporal left |  | 0.491 | 0.051 | -0.105 | 2.613 | -0.272 | 2.378 |
| theta |  |  |  |  |  |  |  |
| inferior parietal |  | 0.859 | 0.041 | -0.028 | 1.351 | -0.283 | 2.447 |
| frontal |  | 0.416 | 0.055 | -0.125 | 1.451 | -0.262 | 2.396 |
| temporal right |  | 0.559 | 0.048 | -0.091 | 1.481 | -0.267 | 2.428 |
| temporal left |  | 0.460 | 0.053 | -0.113 | 1.662 | -0.269 | 2.382 |
| alpha |  |  |  |  |  |  |  |
| inferior parietal |  | 0.755 | 0.042 | -0.051 | 2.292 | -0.271 | 2.548 |
| frontal |  | 0.839 | 0.041 | -0.032 | 2.134 | -0.282 | 2.435 |
| temporal right |  | 0.335 | 0.062 | -0.144 | 1.418 | -0.279 | 2.333 |
| temporal left |  | 0.496 | 0.051 | -0.102 | 1.452 | -0.288 | 2.339 |
| beta |  |  |  |  |  |  |  |
| inferior parietal |  | 0.650 | 0.045 | -0.076 | 2.140 | -0.258 | 2.609 |
| frontal |  | 0.818 | 0.041 | 0.036 | 1.874 | -0.302 | 2.466 |
| temporal right |  | 0.735 | 0.043 | -0.053 | 2.182 | -0.274 | 2.468 |
| temporal left |  | 0.636 | 0.045 | -0.076 | 2.006 | -0.263 | 2.514 |
| gamma |  |  |  |  |  |  |  |
| inferior parietal |  | 0.532 | 0.049 | -0.101 | 2.071 | -0.255 | 2.508 |
| frontal |  | 0.869 | 0.040 | 0.026 | 1.660 | -0.298 | 2.465 |
| temporal right |  | 0.657 | 0.045 | 0.071 | 2.114 | -0.316 | 2.509 |
| temporal left |  | 0.978 | 0.040 | 0.004 | 1.968 | -0.292 | 2.442 |
